# Spatial expression of aquaporin 5 in mammalian cornea and lens, and regulation of Its localization by phosphokinase A

**Published:** 2012-04-18

**Authors:** S. Sindhu Kumari, Murali Varadaraj, Venkata S. Yerramilli, Anil G. Menon, Kulandaiappan Varadaraj

**Affiliations:** 1 Department of Physiology and Biophysics, State University of New York at Stony Brook, Stony Brook, NY; 2 InSTAR Program, Ward Melville High School, East Setauket, NY; 3 Department of Molecular Genetics, Biochemistry and Microbiology, University of Cincinnati College of Medicine, Cincinnati, OH; 4 SUNY Eye Institute, New York, NY

## Abstract

**Purpose:**

Aquaporins (AQPs) play a significant role in the movement of water across the plasma membrane. In the eye, the cornea and lens are avascular with unique microcirculatory mechanisms to meet the metabolic demands. We have previously shown that AQP0 and AQP1 water channels participate in maintaining lens transparency and homeostasis. In the present investigation, we explored the expression and spatial distribution of AQP5 in the cornea and lens, and its regulation during membrane localization.

**Methods:**

AQP5 expression and cellular localization were investigated by reverse transcription polymerase chain reaction (RT-PCR) using gene-specific primers, and by western blot and immunocytochemistry analyses using specific antibodies. AQP5 phosphorylation was studied using calf intestinal alkaline phosphatase for dephosphorylation. Effects of phosphokinase A (PKA) agonist cyclic AMP (cAMP), and antagonist H-89 on AQP5 expression and localization were studied in vitro using MDCK (Madin-Darby Canine Kidney) cells, and ex vivo using isolated corneas from wild type mice.

**Results:**

RT–PCR revealed the presence of AQP5 transcripts in the cornea, lens epithelial cells and fiber cells. Western blotting identified the presence of both non-phosphorylated and phosphorylated forms of AQP5 protein. Immunostaining showed the distribution of AQP5 in the epithelial layer and stromal keratocytes of the cornea, and epithelial and fiber cells of the lens. In vitro and ex-vivo experiments revealed PKA-induced AQP5 internalization; PKA inhibition prevented such internalization.

**Conclusions:**

This is the first report on the spatial expression of AQP5 in the corneal keratocytes and lens epithelial cells, as well as on the regulation of AQP5 localization by PKA in the corneal epithelial cells. PKA-mediated regulation of AQP5 holds promise for therapeutic intervention to control corneal and lens diseases.

## Introduction

The aquaporins (AQPs) are a superfamily of major intrinsic proteins of ~30 kDa, expressed in both prokaryotes and eukaryotes. In mammals, thirteen AQPs have been identified. As in several other organs, water conductance across the many membrane barriers in the eye is assisted by these proteins. Seven AQPs are expressed in the various parts of the eye; three each are present in the mammalian cornea (AQP1, AQP3, AQP5) and lens (AQP0, AQP1, AQP5).

Cornea and lens are avascular tissues with unique microcirculatory mechanisms that are assisted by water channels, for meeting the nutritional demands and removing the metabolic byproducts. In the cornea, the outer stratified epithelium expresses AQP5 and AQP3, stromal keratocytes express AQP1, and the single-celled inner endothelial layer expresses AQP1 and AQP3 [1-3]. In the lens, anterior epithelial cells have AQP1 [3], which functions as a water channel [4,5]. Lens fiber cells abundantly express AQP0 [6] which performs water conductance [4,7], as well as a unique function of cell-to-cell adhesion [8,9].

**Figure 1:**
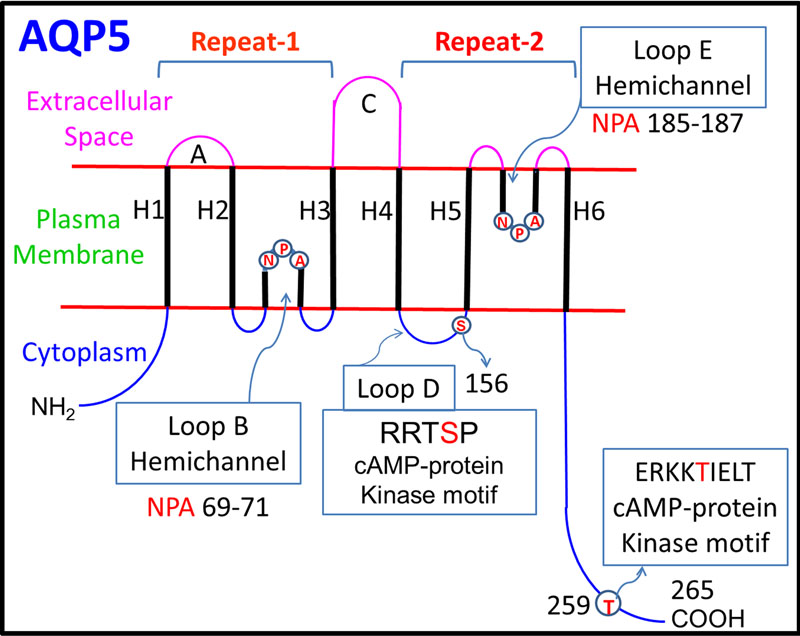
Schematic diagram of mouse AQP5 transmembrane topology. NPA (blue circles) represents the highly conserved aquaporin signature sequence. H1–H6, membrane-spanning helices; A–E, loops; loops B and E form pore helices. NH_2_- and COOH- amino and carboxyl terminal domains, respectively. Two consensus phosphorylation motifs are present, one at amino acid residues RRTSP at 153–157 in loop D and another, RKKT at 256–259 at the COOH-terminal domain.

AQPs contain two tandem repeats (Figure 1), possibly due to gene duplication during evolution. The transmembrane topology of AQP5 shows six membrane-spanning α-helices (H1-H6), and five loops (A-E) that connect the transmembrane domains. Loops B and E act as hemichannels and together form an 'hourglass' structure for water flow; each loop contains a highly conserved, asparagine–proline–alanine (NPA) motif, which is critical for water permeation. Two putative phosphorylation sites [10,11] are present as indicated in Figure 1.

AQP5 is expressed in a wide range of tissues. It is found in lung pneumocyte type I cells [12], granules of Brunner glands in the duodenum [13], in the uterus [14], salivary gland [10,15,16], lacrimal gland [17,18], pancreas [19,20], cornea [1,2,18,21,22], lens [1,23,24], and retina [25,26]. The level of expression is higher in the secretory tissues and glands than in the non-secretory cells. AQP5 plays a significant role in the production of saliva, pulmonary secretions, and tears. After the cloning of AQP5 from rat submandibular gland [10], studies conducted using AQP5 knockout mouse (AQP5-KO) model have corroborated that AQP5 plays an important role in salivary secretion [27,28] and corneal thickness [29]. However, tear secretion was not altered in the AQP5-KO mouse [30,31].

The presence of AQP5 transcripts in the cornea [1] and lens [1,32], and AQP5 protein in the cornea [2,18,22] and lens fiber cells [23,24] has been documented. Patil et al. [1] used reverse transcription polymerase chain reaction (RT-PCR), and Wistow et al. [32] followed expressed sequence tag (EST) analysis to explore the presence of AQP5 transcripts in the lens. Immunocytochemical studies [2,18,22] identified the presence of AQP5 only in the epithelial cells of the cornea. Several other studies of AQP5 protein in the lens used mass spectrometric analysis [23,24], that helps to determine the presence of a particular protein in a sample but does not provide any information on its spatial distribution. Even though the presence of AQP5 protein in the cornea and lens has been identified, there has been no investigation yet to show whether it is expressed overall in these tissues or in specific regions. In the present study, we investigated in detail the expression of AQP5 and its regulation in the cornea and/or lens. Our results showed the presence of AQP5 in the corneal epithelial cells, stromal keratocytes, and in the epithelial and fiber cells of the lens. The study further identified that regulating AQP5 by manipulating phosphorylation could be a tool for therapeutic intervention to control corneal and lens diseases.

## Methods

### Animals

Wild type (WT; C57BL/6) and AQP5 knockout (AQP5-KO [33]) mice, and New Zealand white rabbits were used. Eyes were enucleated and transferred to mammalian physiological saline. Corneas and lenses were dissected out under a microscope. All experimental and animal care procedures were performed according to the ARVO Statement for the Use of Animals in Ophthalmic and Vision Research, and were approved by the State University of New York at Stony Book Institutional Animal Care and Use Committee.

### RT-PCR, cloning and sequencing

Total RNA was extracted separately from mouse lacrimal gland, whole cornea, and lens anterior epithelial cells and fiber cells, using RNA STAT-60 (TEL-TEST Inc., Friendswood, TX). Following the manufacturer's instructions [5], RT-PCR was carried out in a Gradient Thermal Cycler (Stratagene) using *AQP5*-, *AQP0*-, or *AQP1*-specific primers (Table 1) and the products were fractionated on 1.2% agarose gels, stained with ethidium bromide and viewed over UV. Images were captured and digitized using Kodak Image Station [34]. cDNAs amplified from epithelial and fiber RNAs were cloned between the EcoRI and BamHI restriction sites of pIRES2-EGFP vector (Clontech, Mountain View, CA). Transformation was carried out using MAX Efficiency® DH5α™ *E. coli* competent cells (Invitrogen, Life Technologies, Grand Island, NY). Positive clones were selected using kanamycin-Luria-Bertani (LB) agar plates. Sequences of extracted DNAs were verified by automated sequencing using fluorescent dye terminators (Stony Brook University, DNA Sequencing Facility, Stony Brook, NY).

**Table 1:** Primers used for RT-PCR.

**Gene**	**Accession #**	**Primer**	**Sequence**
*AQP5*	BC150769	Forward	5'-ATGAAGAAGGAGGTGTGTTCAG-3'
*AQP5*	BC150769	Reverse	5'-TCAGTGTGCCGTCAGCTCGATGGTC-3'
*AQP0*	NM008600	Forward	5'-ATGTGGGAACTTCGGTCTGCCTC-3'
*AQP0*	NM008600	Reverse	5'-TTACAGGGCCTGAGTCTTCAGTTC-3'
*AQP1*	BC007125.1	Forward	5'-ATGGCCAGTGAAATCAAGAAG-3'
*AQP1*	BC007125.1	Reverse	5'-CTATTTGGGCTTCATCTCCAC-3'

### Western blotting, dephosphorylation, and immunostaining

Total membrane proteins extracted from whole cornea and lens of rabbit and mouse, as well as from mouse lacrimal gland (positive control) and different regions of lens [4] were used for western blotting [34,35]. In short, cornea, lens epithelium, lens cortex and lens nucleus were dissected out under ice-cold physiological saline. Tissues were homogenized using 1× gel loading buffer (Invitrogen, Life Technologies) in 25 mM Tris-HCl (pH 7.2) with proteinase inhibitor cocktail (Sigma) and/or phosphatase inhibitor ( Roche Diagnostics Corporation, Indianapolis, IN). The homogenates were spun at 45,000× g for 30 min and the membrane pellets were washed two times with the homogenizing buffer. The membrane pellets were homogenized in 50–100 μl of protein extraction buffer (2% SDS) and sonicated for 1 min with short bursts. The homogenates were centrifuged at 12,000× g for 30 min, and the supernatants were mixed with NuPAGE LDS sample buffer (Invitrogen) and fractionated using 10% NuPAGE Novex Bis-Tris gel with MOPS-SDS as the running buffer (Invitrogen). Proteins from the denaturing gels were transferred to PVDF nylon membranes and exposed to COOH-terminal-specific anti-AQP5, anti-AQP1 or anti-AQP0 antibodies (Chemicon, Billerica, MA or Santa Cruz Biotechnology, Inc., Santa Cruz, CA) as appropriate. Alkaline phosphatase conjugated secondary antibodies were used and detection of antibody binding was carried out using alkaline phosphatase detection kit (Vector Laboratories, Burlingame, CA).

In dephosphorylation studies, calf intestinal alkaline phosphatase (25 units) was added to ~25 μg of purified membrane protein samples and incubated for 60 min at 37 °C. Reactions were stopped with 50 mM EDTA and analyzed by western blotting as described above [34].

To investigate the spatial expression of different AQPs, eyes of WT and AQP5-KO mice were fixed in 4% paraformaldehyde, cryosectioned, and immunostained with anti-AQP5 (Chemicon, Billerica, MA or Epitomics, Inc., Burlingame, CA), anti-AQP1 or anti-AQP0 antibodies as appropriate, and imaged using a Zeiss confocal microscope as described [5]. Briefly, cornea and in vitro culture cells were fixed in 4% paraformaldehyde in phosphate-buffered saline (PBS) for 30 min; lenses were fixed for 12 h. Fixed corneas and lenses were cryoprotected, and sectioned at 10-16 μm thickness using a cryomicrotome (Leica) and stored at −20 °C. Cryosections, and fixed culture cells were permeabilized (0.2% [v/v] Triton X-100) for 20 min at room temperature, blocked with normal goat serum and treated with rabbit polyclonal antibody raised against AQP5, AQP0, or AQP1, at a dilution of 1:200 with 5% (w/v) bovine serum albumin (BSA) in PBS. After overnight incubation and three washes in PBS, the slides were exposed to fluorescein 5′-isothiocyanate (FITC) or Texas Red conjugated goat anti-rabbit IgG in PBS containing 5% BSA. The slides with the treated tissue sections or cells were washed again in PBS, mounted in anti-fade Vectamount (Vector Laboratories, Burlingame, CA) containing nuclear stain DAPI and viewed. Optimized Z-sectional digital images were acquired and deconvolved using Zeiss AxioVision software Version 5. Representative images are given.

### Effects of cAMP and H-89 on AQP5 expression, trafficking, and localization

Mouse AQP5 expression construct (AQP5-pIRES2-EGFP) was transfected into Madin Darby Canine Kidney (MDCK) cells. MDCK cells were used for in vitro studies on AQP5 expression, trafficking, and localization because these cells do not express AQP5 and are epithelial cells, like the corneal and lens epithelial cells with apical and basolateral sides. In short, MDCK cells (American Type Culture Collection, Manassas, VA) were grown in Minimum Essential Medium (Invitrogen, Life Technologies, Grand Island, NY) that was supplemented with 5% heat-inactivated fetal calf serum (Hyclone Laboratories, Inc., UT) and 100 U/ml penicillin and 0.1 mg/ml streptomycin. Cell cultures were maintained at 37 °C, under 5% CO_2_ in a humidified atmosphere. Transfections were carried out using Effectene reagent (Qiagen), following the manufacturer's recommendations. Stable cell lines were selected using Geneticin® (G418; A.G. Scientific, Inc., CA) Sulfate (400 mg/ml media).

Stable cells expressing AQP5 were exposed to PKA agonist mp-cAMP (membrane permeable cyclic AMP, 100 mM; Sigma-Aldrich, St. Louis, MO) or PKA antagonist H-89 (20 mM; Sigma-Aldrich) for 30 min. In another experiment, cells were incubated first with the H-89 for 30 min, and then stimulated with mp-cAMP for the same duration. Cells were washed in PBS, fixed in 4% paraformaldehyde and immunostained using anti-AQP5 antibody as described above [34].

For ex vivo investigation, WT mouse corneas were incubated in Minimum Essential Medium with 2% fetal bovine serum containing the PKA agonist or antagonist for 30 min or 6 h. Corneas were washed with PBS, fixed, cryosectioned, and immunostained using anti-AQP5 antibody as described above [5].

## Results and Discussion

### Expression of AQP5 transcripts in the mouse cornea and lens

**Figure 2:**
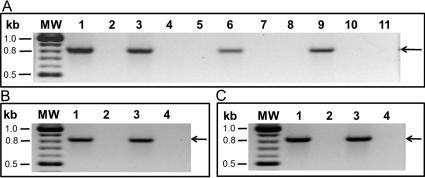
Reverse-transcription polymerase chain reaction (RT–PCR) analysis of AQP5. **A**: In mouse lens. Lanes: 1 Wild type (WT) lacrimal gland (positive control), 2. AQP5 knockout (AQP5-KO) lacrimal gland, 3. WT cornea, 4. WT cornea + RNase, 5. AQP5-KO cornea, 6. WT lens epithelium, 7. AQP5-KO lens epithelium, 8. WT lens epithelium + RNase, 9. WT lens cortex, 10. WT lens cortex + RNase, 11. AQP5-KO lens cortex, MW-Molecular weight marker. **B**: RT–PCR analysis of AQP1 in mouse lens epithelial cells. Lanes: 1. WT lens epithelium, 2.WT lens epithelium + RNase, 3. AQP5-KO lens epithelium, 4. AQP5-KO lens epithelium + RNase, MW-Molecular weight marker. **C**: RT–PCR analysis of AQP0 in mouse lens fiber cells. Lanes: 1. WT lens cortex, 2. WT lens cortex + RNase, 3. AQP5-KO lens cortex, 4. AQP5-KO lens cortex + RNase, MW-molecular weight marker.

Total RNA samples from the WT and AQP5-KO mice were subjected to RT–PCR to determine the expression of AQP5, AQP1, or AQP0 using gene-specific primers (Table 1), at the message level. Figure 2A shows the amplification of an approximately ~0.8 kb DNA segment from the WT lacrimal gland (positive control), cornea, lens epithelium, and lens cortical fiber cell samples (lanes 1, 3, 6, and 9, respectively). As expected, comparable samples of the AQP5-KO mouse (lanes 2, 5, 7, and 11, respectively) did not show any amplification. *AQP5* genomic DNA sequence has three introns and four exons. The total RNAs prepared were not contaminated with the genomic DNA, since the RT–PCR-amplified products showed a single band of ~0.8 kb (Figure 2A, lanes 1, 3, 6, and 9) comparable to the PCR product of cloned *AQP5* cDNA (798 bp; not shown). In addition, there was no amplification from the RNase I treated WT RNAs (Figure 2A, lanes 4, 8, and 10). To confirm that the transcripts were those of AQP5, we cloned and sequenced the RT–PCR products of mouse lens epithelial cells, and fiber cells. FASTA analyses showed 100% homology to the mouse *AQP5* sequence in the GenBank (BC150769) verifying the authenticity of the identified *AQP5* transcripts.

Figure 2B shows the expression of *AQP1* transcripts in the epithelial cells of WT and AQP5-KO mice (lanes 1 and 3) and their absence in the RNase-treated samples (lanes 2 and 4). Similarly, *AQP0* transcripts were present in the lens fiber cells of WT and AQP5-KO mice (Figure 2C, lanes 1 and 3), and were not seen when treated with RNase (lanes 2 and 4). In summary, RT–PCR data for lens revealed the presence of *AQP5* and *AQP1* transcripts in the epithelial cells and *AQP5* and *AQP0* transcripts in the fiber cells.

### Expression of AQP5 protein in the cornea and lens

The presence of AQP5 expression at the message level in mouse cornea and lens prompted us to find out whether there is expression at the protein level. Information about the cellular and subcellular localization of AQP5 protein is critical for understanding the role of AQP5 in these tissues. Therefore, we performed western blotting and immunostaining.

**Figure 3:**
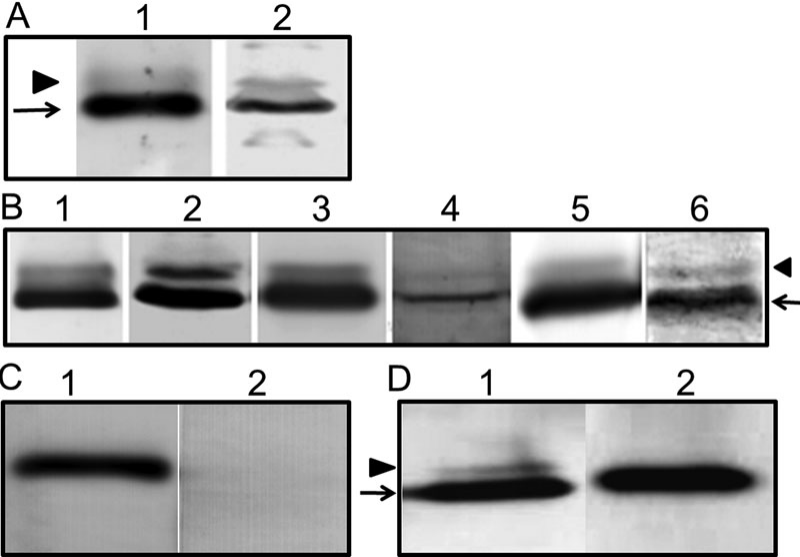
Immunoblot analyses of corneal and lens membrane proteins to identify the expression of AQP5. **A**: From rabbit: Lanes; 1. total cornea, 2. total lens. **B**: From mouse: Lanes: 1. lacrimal gland (+ve control), 2. total cornea, 3. total lens, 4. lens epithelial cell membrane, 5. lens cortex fiber cell membrane, 6. lens nuclear fiber cell membrane. **C**: AQP5-KO mouse lens fiber cell membrane proteins. Lanes: 1. AQP0, 2. AQP5, expressions studied using anti-AQP0 and anti-AQP5 antibodies, respectively. **D**: Immunoblot of dephosphorylation studies in WT corneal membrane proteins using anti-AQP5 antibody. Lanes: 1. WT untreated proteins (arrow - ~28 kDa; arrowhead - ~34 kDa), 2. WT proteins treated with calf intestinal alkaline phosphatase; the 34 kDa band disappeared, presumably due to dephosphorylation.

Total membrane proteins from rabbit cornea and lens were immunoblotted with anti-AQP5 antibody (Figure 3A, lanes 1 and 2) which bound to two bands of ~28 (arrow) and ~34 kDa (arrowhead). Similar binding occurred with samples of mouse lacrimal gland (positive control; Figure 3B, lane 1), cornea (lane 2), lens (lane 3) and distinct regions of the lens (lanes 4–6). Total membrane proteins from AQP5-KO mouse lens immunoblotted with anti-AQP0 (Figure 3C, lane 1) and anti-AQP5 (Figure 3C, lane 2) antibodies separately, showed the expression of AQP0 and the absence of AQP5, respectively, substantiating the reaction specificity. In Figure 3A, the ~28 kDa band (arrow) corresponds to the unmodified AQP5 while the ~34 kDa band (arrowhead) could be due to post-translational modification such as phosphorylation. AQP5 has two consensus PKA/PKG-activated phosphorylation motifs (Figure 1), one at residues 153–157 (RRT**S**P) in loop D, and the second at residues 256–259 (RKK**T**) at the COOH-terminal domain. To test whether the 34 kDa band could be a phosphorylation product, we treated mouse corneal membrane proteins (Figure 3D, lane 1) with calf intestinal alkaline phosphatase. The ~34 kDa band disappeared (Figure 3D, lane 2) presumably, due to dephosphorylation.

To date, AQP1 in the epithelial cells and AQP0 in the fiber cells, have been functionally characterized in vitro using *Xenopus* oocytes and ex vivo using lens epithelial cells and fiber cell vesicles [4,5,9,34,36-38]. Published data on the expression of AQP5, in the lens are conflicting. Trace amounts of *AQP5* transcript had been documented for rat [1] and dog [39] lens tissues. However, immunoblotting of rabbit [40] and dog [39] lens fiber cell membrane proteins did not detect AQP5. Mass spectrometry studies showed the presence of AQP5 among bovine [23] and murine [24] total fiber cell membrane proteins. We performed immunocytochemical studies to distinctly define the presence and localization of AQP5 within the specific regions of mouse cornea and lens.

**Figure 4:**
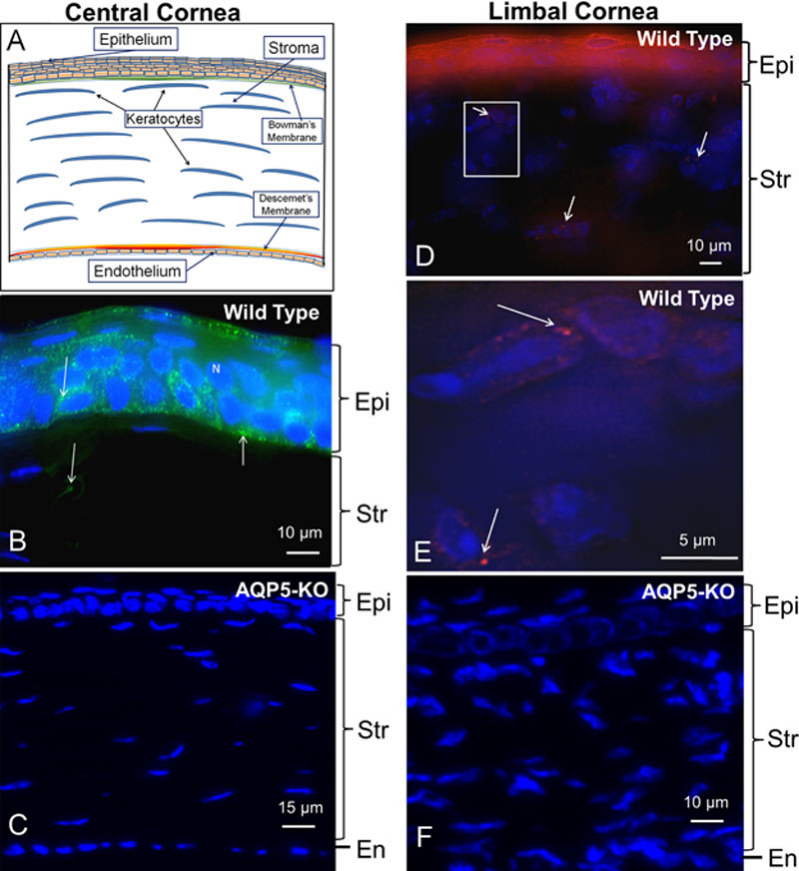
Immunolocalization of AQP5 in mouse cornea. **A**: Schematic diagram of a mammalian cornea showing the five layers. **B**: AQP5 localization (green) in central corneal epithelial cells and stromal keratocytes. **C**: AQP5 knockout mouse central corneal epithelial cells and stromal keratocytes showing lack of immunoreactivity. **D**: AQP5 localization (red) in the limbal area of the cornea; the window in **D** is enlarged and shown as **E**. **E**: AQP5 (red) in limbal stromal keratocytes. **F**: AQP5 knockout mouse corneal stromal keratocytes in the limbal area with no immunoreactivity. **B**, **C**: FITC conjugated secondary antibody. **D**, **E**, **F**: Texas Red conjugated secondary antibody; blue, nuclear stain DAPI. Epi: epithelium; Str: stroma; En: endothelium; arrows- antibody binding.

Most mammalian corneas have five layers (Figure 4A) from anterior to posterior [41,42]: corneal epithelium, bowman's layer, stroma, descemet's membrane, and endothelium. AQP5 expression and localization have been reported only for the epithelial cell layer and not for other corneal cell layers in the WT mouse [43], rat [22] and dog [39]. Figure 4B shows that in the mouse cornea, AQP5 is expressed in the outermost multilayered epithelial cells, single layered basal columnar epithelium (abundant expression) and in the suprabasal cells. Low level expression is seen in the multilayered flat polygonal cells. Figure 4C corroborates the specificity of antibody binding by the lack of immunoreactivity in the AQP5 knockout mouse cornea. In the stroma, AQP5 is expressed at low levels in keratocytes close to Bowman’s membrane in the central cornea (Figure 4B), and at high levels in keratocytes at the limbal area (Figure 4D and E) which is the junction of the cornea and the conjunctiva. There is no expression of AQP5 in the other parts of the cornea including the endothelial cells. Immunostaining of cryosections of AQP5 knockout limbal area (Figure 4F) showed no antibody binding, validating the specificity of immunoreactions in the WT cornea.

AQP5 in the corneal epithelial cells and keratocytes could be involved in preventing corneal dehydration, promoting wound healing and maintaining corneal transparency. AQP5-KO mice showed significant reduction in corneal epithelial cell membrane water permeability (~2 fold) and increase in corneal thickness [29]. When corneal epithelial cells were subjected to hypotonic conditions, the recovery rate after swelling was significantly reduced in AQP5-KO compared to the WT mice [29]. The corneal epithelial layer serves as a barrier against pathogenic invasion and helps to keep the cornea moist. In the epithelial cells, AQP5 may be critical for corneal fluid dynamics; malfunction can cause corneal edema and dry eye disease [43,44]. Corneal edema can lead to loss of transparency. Kenney et al. [45] analyzed AQPs in normal, diseased and cataract-post-surgical corneas and observed AQP expression abnormalities under several disease conditions.

**Figure 5:**
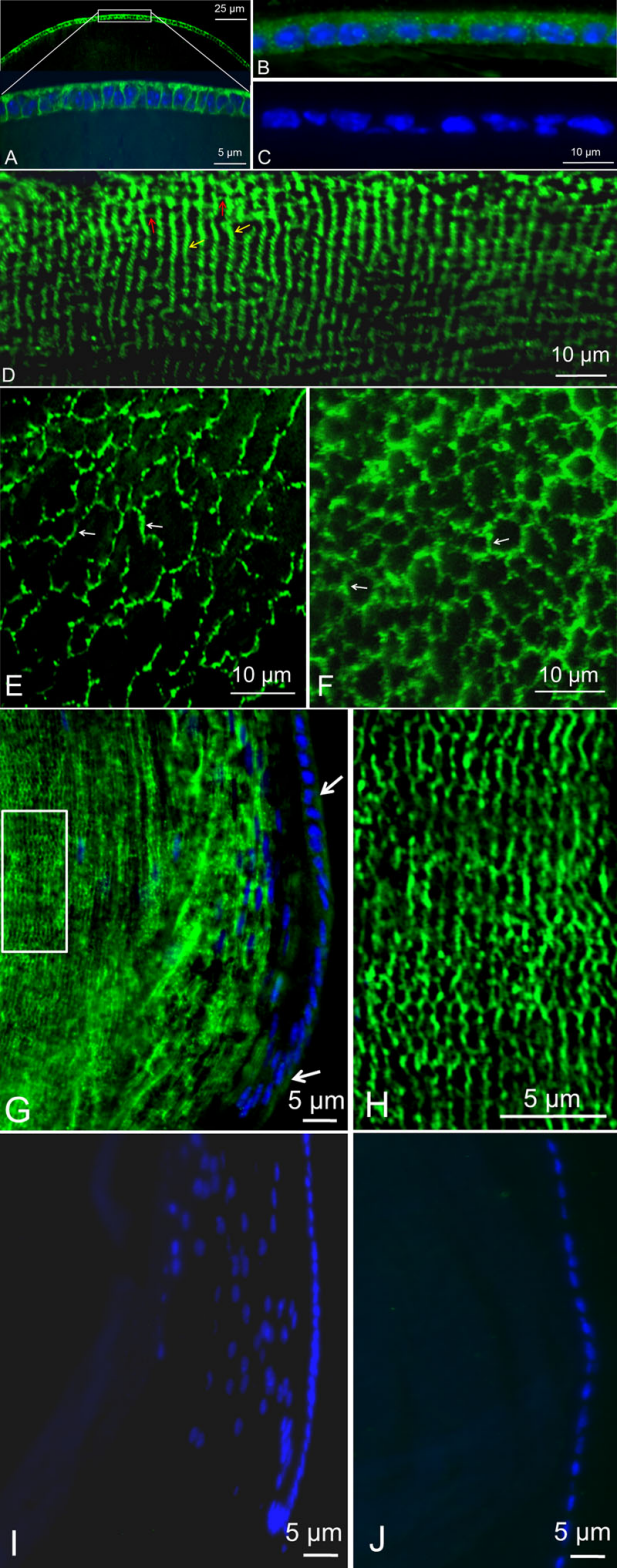
Immunolocalization of AQP5 protein in comparison with AQP1 or AQP0 in mouse lens. **A**: AQP1 expression in the WT lens anterior epithelial cells; a window of anterior epithelial cells is enlarged and shown below, in the same figure. **B**: AQP5 expression in the WT lens epithelial cells. **C**: (negative control), AQP5 knockout mouse lens section showing lack of immunoreactivity in the epithelial cells. **D**: WT lens outer cortex fiber cells with AQP5 expression. **E**: WT lens inner cortex fiber cells with AQP5 expression. **F**: AQP0 expression in the lens inner cortex fiber cells (very intense immunoreactivity compared to AQP5 expression shown in **E**). **G**: WT lens equatorial region showing AQP5 in the epithelial and fiber cells. **H**: The window shown in **G** is enlarged to provide a clear view of anti-AQP5 antibody binding to the narrow end of the fiber cells. **I**, **J**: AQP5 knockout mouse lens sections showing lack of immunoreactivity in the cells at the equatorial and anterior regions, respectively. **A**-**J**: FITC conjugated secondary antibody; green, antibody binding indicating AQP expression; blue, nuclear stain DAPI. White arrows – antibody binding. Yellow arrows – narrow side of the fiber cell; Red arrows – broader side of the fiber cell; **A**-**C** and **G**-**J** Sagittal sections; **D**-**F**: Cross sections.

In the WT lens epithelial cells, anti-AQP1 antibody bound more intensely to the basolateral side than to the apical side (Figure 5A) while anti-AQP5 antibody bound uniformly throughout with punctate membrane localization (Figure 5B). In AQP5-KO mouse, there was no antibody binding to the epithelial cells (Figure 5C), substantiating the specificity of anti-AQP5 antibody binding in the WT (Figure 5B). Figures 5D and E exhibit the expression of AQP5 in the outer cortex and inner cortex fiber cells, respectively. In Figure 5D, antibody binding is more intense on the narrow sides (yellow arrows) of the fiber cells than on the broader sides (red arrows). In addition, it is apparent that the intensity of antibody binding gradually decreases as the fiber cells run deep toward the nucleus of the lens. This difference in antibody binding could be due to epitope masking or loss of epitopes due to fiber cell aging when the cells lose their COOH-terminal ends; the antibodies tested were raised against COOH-terminal epitopes. We have previously reported a similar decrease in antibody affinity in the fiber cells, with regard to anti-AQP0 antibody binding [37]. AQP5 is expressed at low levels in both lens epithelial (Figure 5B) and fiber cells (Figure 5D,E) compared to AQP1 (Figure 5A) and AQP0 (Figure 5F) in the respective cells types. Epithelial and fiber cells in the equatorial region also bound the anti-AQP5 antibody (Figure 5G); the window in Figure 5G is enlarged (Figure 5H) to show the immunoreactivity to the fiber cells. As expected, the equatorial (Figure 5I) and anterior (Figure 5J) regions of the AQP5-KO mouse lens sections showed no antibody binding.

It is estimated that AQP0 constitutes ~44.8% of the total lens fiber cell membrane protein in contrast to AQP5 that constitutes only ~0.36% [24]. In mammalian lens, AQP0 contributes ~80% of the water permeability [4,36,46] in the lens fiber cells even though it is >40-fold less efficient than AQP1 [47]. Mouse lens fiber cell lipid contributes only ~1.5% of the membrane water permeability due to the high levels of cholesterol and sphingomyelin [4,46,48]. We speculated [4] that ~18.5% of the fiber cell water permeability may be contributed by the co-transporters. The present study suggests that the 18.5% water permeability could come from AQP5 itself, or in combination with the membrane co-transporters. Lately, passage of water through fiber cell gap junctions has been postulated [49]. Even though, the expression level is ~100 fold less than AQP0 [24], the single channel water permeability of AQP5 is ~20 fold higher [50]. However, in the AQP0 knockout mice, the compensatory water permeability wielded by AQP5 appears insufficient since the animals developed cataract. In addition, the cell-to-cell adhesion capability could be unique to AQP0; crystallographic structural data [51] do not support the possibility of such a function for AQP5. In the WT, AQP5 could be complementing the water permeability needs of the fiber cells along with AQP0. Any additional function for AQP5 other than water permeability remains unclear. Lack of AQP1 in the lens epithelial cells of human [52] or mouse [53] did not cause lens abnormalities, opacity or cataract. However, AQP1 knockout mouse lenses subjected to stressful conditions, developed lens opacity/cataract with in vitro and in vivo models [53]. Under normal conditions, AQP5 present in the epithelial cells may fulfill the water permeability function in the AQP1 knockout lens, but apparently not under stressful conditions. Despite the nonoccurrence of phenotypes (based on the gross lens morphology) due to the absence of AQP5 in the knockout mouse, it cannot be ruled out that the function/s of AQP5 could be irrelevant, until more details emerge from a wide range of studies including the structural and functional aspects of this protein.

In the lens, while AQP1 is present in the epithelial cells and AQP0 is present in the fiber cells, AQP5 has a global expression. It is reasonable to speculate that the highly efficient AQP5 water channel may aid in maintaining lens transparency and homeostasis. Using AQP0, AQP1 and AQP5-KO mouse models, currently we are investigating the role of AQP5 in maintaining lens transparency and homeostasis.

### Regulation of AQP5 localization

**Figure 6:**
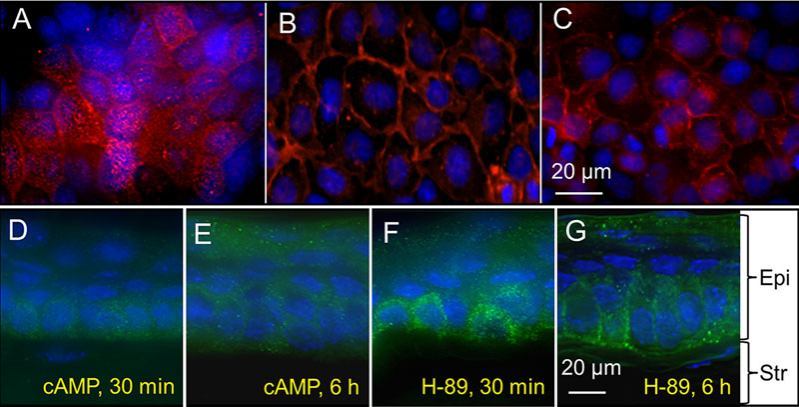
Effects of PKA activator mp-cAMP or PKA inhibitor H-89 on AQP5 protein localization. MDCK cells expressing AQP5 were exposed to: mp-cAMP (**A**); H-89 (**B**); H-89 first and then stimulated with mp-cAMP (**C**). **D**-**G**: Mouse corneal epithelial AQP5 regulation studies in organ culture. Mouse eyes were dissected out and cultured in MEM containing 2% FBS with mp-cAMP (**D**, **E**) or H-89 (**F**, **G**) for 30 min (**D**, **F**) or 6 h (**E**, **G**). MDCK cells expressing AQP5 (**A**-**C**) and cryosections of cultured eyes were immunostained for corneal AQP5 expression (**D**-**G**). Epi: epithelium; Str: stroma.

Several investigators have demonstrated that AQP5 expression, intracellular trafficking and localization in the epithelial cells are regulated by cAMP through the PKA pathway [54-56]. In our in vitro studies, plasma membrane localization of AQP5 decreased significantly when MDCK cells expressing mouse AQP5 were exposed to mp-cAMP (100 μM), a PKA agonist, for 30 min (Figure 6A). On the contrary, when the cells were exposed to a PKA antagonist, H-89 (20 μM) for the same duration, there was increase in AQP5 plasma membrane localization and abundance (Figure 6B). Cells that were incubated first with the PKA inhibitor H-89 for 30 min and then stimulated with mp-cAMP for 30 min, did not show significant AQP5 internalization (Figure 6C) as opposed to cells exposed only to mp-cAMP (Figure 6A). Similarly, in ex-vivo experiments, plasma membrane localization of AQP5 decreased significantly when WT corneas were exposed to mp-cAMP (100 μM) for 30 min or 6 h (Figure 6D,E). When corneas were exposed to the PKA inhibitor, H-89 (20 μM) for 30 min or 6 h there was increase in AQP5 plasma membrane localization and abundance (Figure 6F,G) depending upon the extent of exposure.

AQP5 has several potential phosphorylation sites (Figure 1). Two consensus PKA/PKG-activated phosphorylation motifs are present at residues RRTSP at 153–157 and RKKT at 256–259, in loop D and at the COOH-terminal domain, respectively. Sidhaye et al. [55] had shown cAMP-induced biphasic effect on AQP5 expression and membrane localization when cells expressing AQP5 were exposed to cAMP for short and long-terms. AQP5 protein internalization, lysosomal degradation and a subsequent decrease in AQP5 protein in the plasma membrane occurred in mouse lung epithelial cells exposed to cAMP for a short-term, but long-term exposure resulted in increased abundance and an increase in trafficking of AQP5 to the plasma membrane. Expression and localization of AQP5 in mouse lung epithelial cell line (MLE-12) were regulated at transcriptional and posttranscriptional levels, respectively [54]. Recently, Hasegawa et al. [56] reported that AQP5 is phosphorylated at Thr259 by PKA through cAMP. Based on the current investigation and previous studies, we hypothesize that dynamic regulation of AQP5 expression and localization through PKA pathway could be a potential tool for short and long-term regulation of membrane water permeability in the cornea and lens. During aging, when most of the lens proteins including aquaporins undergo modifications leading to alterations and/or loss of function, increasing the availability of AQP5 water channels in the plasma membrane by dephosphorylating the AQP5 present in the sub-cellular pool using small molecular pharmacological compound/drugs could augment membrane water permeability to maintain lens microcirculation and homeostasis. Further investigation of this regulatory pathway may provide a strong base for developing novel therapeutic drug targets to treat corneal diseases and lens cataracts.

In conclusion, we have defined the spatial distribution pattern of AQP5 in the cornea and lens. In the cornea, AQP5 is expressed in the stromal keratocytes in low levels compared to the epithelial layer. In the lens, AQP5 is expressed in the epithelial and fiber cells. AQP5 may play a significant role in maintaining transparency and homeostasis in the avascular cornea and lens. A thorough understanding of the functional complementation or uniqueness of the corneal and lens AQPs will facilitate development of therapeutic intervention strategies to avert pathological corneal swelling and opacity, as well as age-onset lens opacity and cataract.
